# A comparative profile of total protein and six angiogenically-active growth factors in three platelet products

**DOI:** 10.3205/iprs000167

**Published:** 2022-07-05

**Authors:** Scott Custo, Byron Baron, Alex Felice, Elisa Seria

**Affiliations:** 1 Department of Physiology & Biochemistry, Faculty of Medicine & Surgery, University of Malta, Msida, Malta; 2 Centre for Molecular Medicine & Biobanking, University of Malta, Msida, Malta; 3 Division of Clinical Genetics, Department of Pathology, Mater Dei Hospital, Msida, Malta

**Keywords:** wound healing, platelet-derived products, growth factors, platelet lysate (PL), platelet-rich plasma (PRP), platelet-rich fibrin (PRF)

## Abstract

**Objectives:**

Platelet-derived products have been shown as promising novel therapeutic agents for chronic wounds. However, their clinical use requires a greater degree of method standardisation, part of which involved more extensive cataloguing of their biochemical composition. This study aimed to quantify and compare total protein and 6 angiogenically-active growth factors in three distinct platelet products.

**Methods:**

Platelet Lysate (PL, n=5), Calcium-activated Platelet Rich Plasma (Ca-PRP, n=5) and Platelet-Rich Fibrin (PRF, n=5) were prepared from pooled platelet apheresis products (n=10). Ca-PRP and PRF were prepared from the same units (n=5) by activation with 20 mmolL^-1^ calcium chloride. PL was prepared from the remaining (n=5) units using an established lysate. Total protein was quantified with the Bradford Assay. Sandwich enzyme-linked immunosorbent assay was used to quantify six growth factors: epidermal growth factor (EGF), vascular endothelial growth factor (VEGF), hepatocyte growth factor (HGF), stromal cell derived growth factor-1α (SDF-1α), endostatin, and transforming growth factor-β1 (TGF-β1).

**Results:**

Protein retrieval differed significantly (p<0.05) between the three products: PL (11.35±0.80 mg/mL) < Ca-PRP (20.44±8.17 mg/mL) < PRF (40.67±3.13 mg/mL). Growth factor yield was considerable in all three products and differed significantly for: VEGF (PL<PRF); EGF (Ca-PRP<PRF); HFG (PL<Ca-PRP); Endostatin (PL<Ca-PRP, PRF<Ca-PRP, PL<PRF) and TGF-β1 (Ca-PRP<PL, Ca-PRP<PRF).

**Conclusions:**

Platelet apheresis products contain a substantial quantity of the investigated pro- and anti-angiogenic growth factors. Their release varies depending on the manufacturing protocol used. Clinically, alternate products could thus be combined to provide a therapeutically optimal mix of growth factors.

## Zusammenfassung

**Zielsetzung:**

Aus Blutplättchen gewonnene Produkte haben sich als vielversprechende neue Therapeutika für chronische Wunden erwiesen. Ihre klinische Verwendung erfordert jedoch ein höheres Maß an Standardisierung der Methoden, wozu auch eine umfassendere Katalogisierung ihrer biochemischen Zusammensetzung gehört. Ziel dieser Studie war es, das Gesamtprotein und 6 angiogen aktive Wachstumsfaktoren in drei verschiedenen Thrombozytenprodukten zu quantifizieren und zu vergleichen.

**Methoden:**

Thrombozytenlysat (Platelet Lysate PL, n=5), kalziumaktiviertes plättchenreiches Plasma (Calcium-activated Platelet Rich Plasma Ca-PRP, n=5) und plättchenreiches Fibrin (Platelet-Rich Fibrin PRF, n=5) wurden aus gepoolten Thrombozytenaphereseprodukten (n=10) hergestellt. Ca-PRP und PRF wurden aus denselben Einheiten (n=5) durch Aktivierung mit 20 mmolL^-1^ Calciumchlorid hergestellt. PL wurde aus den verbleibenden (n=5) Einheiten unter Verwendung eines etablierten Lysats hergestellt. Das Gesamtprotein wurde mit dem Bradford-Assay quantifiziert. Mit dem Sandwich Enzyme-linked Immunosorbent Assay wurden sechs Wachstumsfaktoren quantifiziert: Epidermaler Wachstumsfaktor (Epidermal Growth Factor EGF), vaskulärer endothelialer Wachstumsfaktor (Vascular Endothelial Growth Factor VEGF), Hepatozyten-Wachstumsfaktor (Hepatocyte Growth Factor HGF), CXCL12 (Stromal Cell Derived Growth Factor-1α SDF-1α), Endostatin, transformierender Wachstumsfaktor-β1 (Transforming Growth Factor-β1 TGF-β1).

**Ergebnisse:**

Die Proteinausbeute unterschied sich signifikant (p<0,05) zwischen den drei Produkten: PL (11,35±0,80 mg/ml) < Ca-PRP (20,44±8,17 mg/ml) < PRF (40,67±3,13 mg/ml). Die Ausbeute an Wachstumsfaktoren war bei allen drei Produkten beträchtlich und unterschied sich signifikant für: VEGF (PL<PRF); EGF (Ca-PRP<PRF); HFG (PL<Ca-PRP); Endostatin (PL<Ca-PRP, PRF<Ca-PRP, PL<PRF) und TGF-β1 (Ca-PRP<PL, Ca-PRP<PRF).

**Schlussfolgerungen:**

Thrombozyten-Apherese-Produkte enthalten eine beträchtliche Menge der untersuchten pro- und antiangiogenen Wachstumsfaktoren. Ihre Freisetzung variiert je nach dem verwendeten Herstellungsprotokoll. Klinisch könnten daher verschiedene Produkte kombiniert werden, um eine therapeutisch optimale Zusammenstellung der Wachstumsfaktoren zu erhalten.

## Introduction

Chronic wounds (CWs) do not progress through the four stages of wound healing in a timely manner, and as such do not heal to a satisfactory degree or within the expected time frame as matched to a comparable acute wound [1]. They are a great cause of physical and psychological morbidity for both patients and families, and are a heavy burden on health systems’ resources [2]. Case in point, in the national health service of the United Kingdom, compared to acute wounds, CWs incurred a mean per-patient rise of:

- 28% in outpatient visits,
- 47% in family doctor visits,
- 100% in prescriptions,
- 178% in wound care products and
- 70%, 162%, and 260% in practice, community and specialist nurse visits, respectively.

Fiscally, this translated into an approximate increase of 63.82%, with the cost rising further for non-healing wounds, whether chronic or not [3]. 

Moreover, total healing of CWs is often difficult or impossible, and they are often plagued with further complications such as scars, or subclinical and diagnosed infections [4], [5]. Indeed, the rate of recurrence and severe infection is high [1]. In fact, in the span of a year, the average rate of CW healing in the national health service (U.K.) between 2012/2013 and 2017/2018, was around 43 to 49% [3], [6], [7], [8]. The rate dropped significantly with diagnosed or suspected infection: 59% (no infection) versus 45% (infection) of CWs [8].

There is thus a clear demand for novel therapeutics. Platelet-derived products (PDPs) have emerged as strong contenders, with several studies demonstrating their effectiveness for CWs and dental and musculoskeletal events [9], [10], [11], [12], [13], [14], [15], [16], [17]. The effect is due to platelets’ wealth of bioactive molecules, chiefly released from their alpha granules such as immunoglobulins and growth factors (GFs) capable of modulating each stage of wound healing [9], [10], [11], [12], [13], [14], [15], [16], [17], [18], [19], [20], [21], [22], [23]. PDPs may therefore be applied to wounds, to stave off and quell infection (IgG content), and simultaneously promote tissue healing through the physiological release of GFs.

Since angiogenesis is a key step in any healing process, angiogenic factors are of particular interest. They are abundant in PDPs and potentially useful for wound therapy. Prominent examples include the pro-angiogenic epidermal growth factor (EGF), vascular endothelial growth factor (VEGF), hepatocyte growth factor (HGF), stromal cell derived growth factor-1α (SDF-1α), and transforming growth factor-β1 (TGF-β1), and the anti-angiogenic endostatin [24], [25], [26], [27], [28], [29], [30], [31], [32], [33]. 

In this context, we quantified six angiogenic growth factors (listed above) in three different PDPs: platelet lysate (PL), calcium-activated platelet-rich plasma (Ca-PRP), and platelet-rich fibrin (PRF), to establish if any statistically significant difference in GF concentration – and thus, therapeutic potency – exists between them. 

## Methods

### Samples

**Figure 1:**
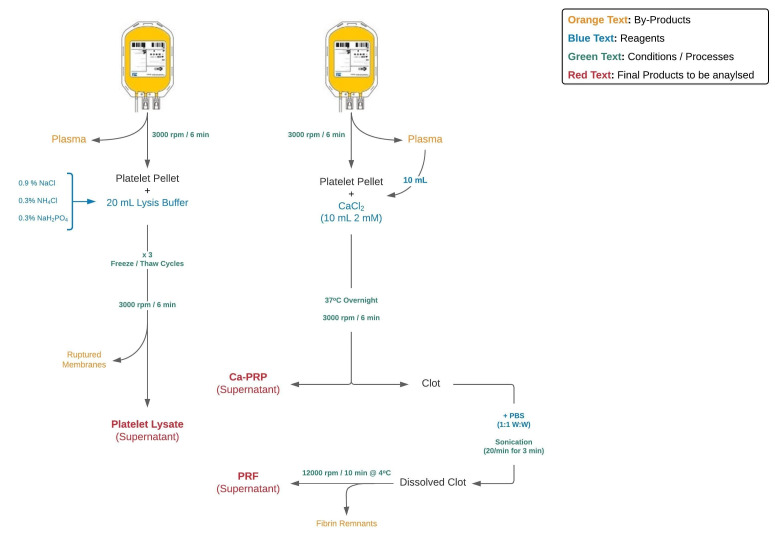
Workflow for sample preparation

The workflow for the preparation of the three PDPs is shown in Figure 1 (Fig. 1).

All PDPs were prepared from five-days-old apheresis-pooled, leukocyte-depleted platelet bags (n=10). Buffy coats for preparing these units were obtained from routine processing of whole blood donated by healthy volunteers at the National Blood Transfusion Center, Guardamangia, Malta. The blood bags allow for natural oxygen / carbon dioxide exchange and were kept at constant agitation up to the point of processing into PDPs.

In all cases, the first step of processing was centrifugation at 2,500 rpm for 6 min, to separate the platelets (pellet) from plasma (supernatant).

#### Platelet lysate (PL)

All supernatant (plasma) was discarded, and the pellet vigorously shaken with 20 mL of lysis buffer (0.9% sodium chloride, 0.3% ammonium chloride and 0.3% sodium dihydrogen phosphate; Sigma-Aldrich, Munich, Germany), modified by Seria et al. [22]. 

Following three consecutive freeze-thaw cycles, a second centrifugation (2,000 rpm for 6 min) was performed to precipitate and remove broken platelet membranes. The supernatant (PL) was aliquoted and stored at –20°C until needed.

#### Calcium-activated platelet-rich plasma (Ca-PRP)

All supernatant (plasma) was removed from the pellet. 10 mL of plasma was replaced to produce PRP, to which was added 10 mL of 20 mmolL^-1^ calcium chloride to activate the platelets. This was left in a 37°C water bath overnight to allow for clotting, and separated by centrifugation at 2,000 rpm for 6 min. The supernatant (Ca-PRP) was aliquoted and stored at –20°C until needed. 

#### Platelet-rich fibrin (PRF)

The clots (PRF) from the above step were re-suspended in phosphate-buffered saline (PBS) in a 1:1 (W:V), PRF:PBS ratio; then broken up via sonication at a frequency of 20 Hz for 10 seconds, 30 Hz for 30 seconds, and 50 Hz for 10 seconds as modified (to prevent heat denaturing) by Lee et al. [34].

The remaining insoluble fibrin was removed by centrifuging at 12,000 rpm for 10 min at 4°C [34]. The supernatant was aliquoted and stored at –20°C until needed.

### Determining total protein content: Bradford assay

Total protein content was determined using the Bio-Rad Bradford protein assay (Bio-Rad Laboratories, CA, USA). 

Standards were set up using varying concentrations of bovine serum albumin (BSA), and PBS as the blank. All samples were performed in duplicate. Both samples and standards were read in triplicate at an optical density (OD) of 595 nm on an Eppendorf BioPhotometer 6131 (Hamburg, Germany). The standard curve was plotted using Microsoft Excel 2016.

### Sodium dodecyl sulfate-polyacrylamide gel electrophoresis (SDS-PAGE)

PL (n=5), Ca-PRP (n=4) and PRF (n=4) were first denatured by heating at 95°C for 5 min with Laemmlli buffer (Bio-Rad, CA, USA).

Precision Plus Protein™ (Bio-Rad, CA, USA) protein ladder and 20 µg of each sample (in the order given above) were loaded into separate lanes of an 8% and 12% polyacrylamide gel. 

After running, both gels were stained with Coomassie Blue (SimplyBlue™ SafeStain, Invotrogen, MA, USA). The 8% gel was stained a second time using silver stain, to better highlight faint bands.

### Sandwich enzyme-linked immunosorbent assay (sELISA)

sELISA (Thermo Fisher Scientific, MA, USA) assays for EGF, VEGF, HGF, SDF-1α, endostatin, and TGF-β1 were conducted on the PL, Ca-PRP and PRF preparations – following the manufacturer’s protocol.

The plates were read at an OD of 450 nm on a Mithras LB940 multimode microplate reader. Blanks were prepared according to the same protocols, and four parameter standard curves were plotted using GraphPad Prism version 9.2.0 (San Diego, CA, USA). All samples (n=5+5+5) were run in duplicate and read in triplicate, giving 30 readings per set of samples.

### Statistical analysis

All values are reported as the mean (± standard deviation) of five samples performed in duplicate and read in triplicate. Data was analysed using GraphPad Prism, version 9.2.0 (San Diego, CA, USA) and SPSS, version 27 (Chicago, IL, USA). Significance analysis of data was performed via one-way analysis of variance (1W-ANOVA), followed by Tukey’s post hoc test for sample group comparisons. Significance was set at p≤0.05.

## Results

**Figure 2:**
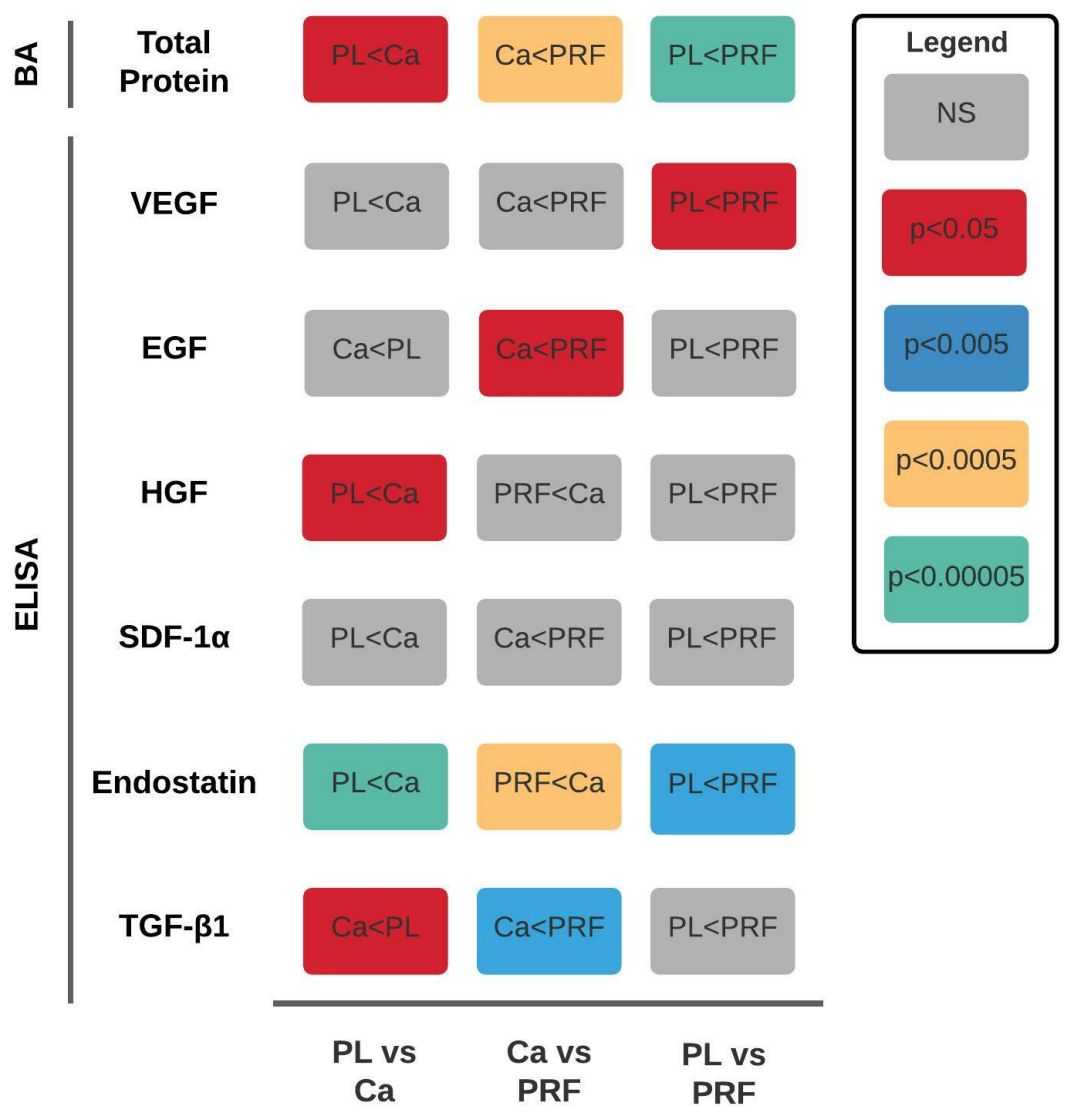
Summary of results. PL, platelet lysate | Ca, calcium-activated platelet-rich plasma | PRF, platelet-rich fibrin | BA, Bradford assay | ELISA, enzyme-linked immunosorbent assay | VEGF, vascular endothelial growth factor | EGF, endothelial growth factor | HGF, hepatocyte growth factor | TGF-β1, transforming growth factor-beta 1 | SDF-1α, stromal cell-derived growth factor-1 alpha | NS, not significant

The results are summarized in Figure 2 (Fig. 2).

### Total protein content

**Figure 3:**
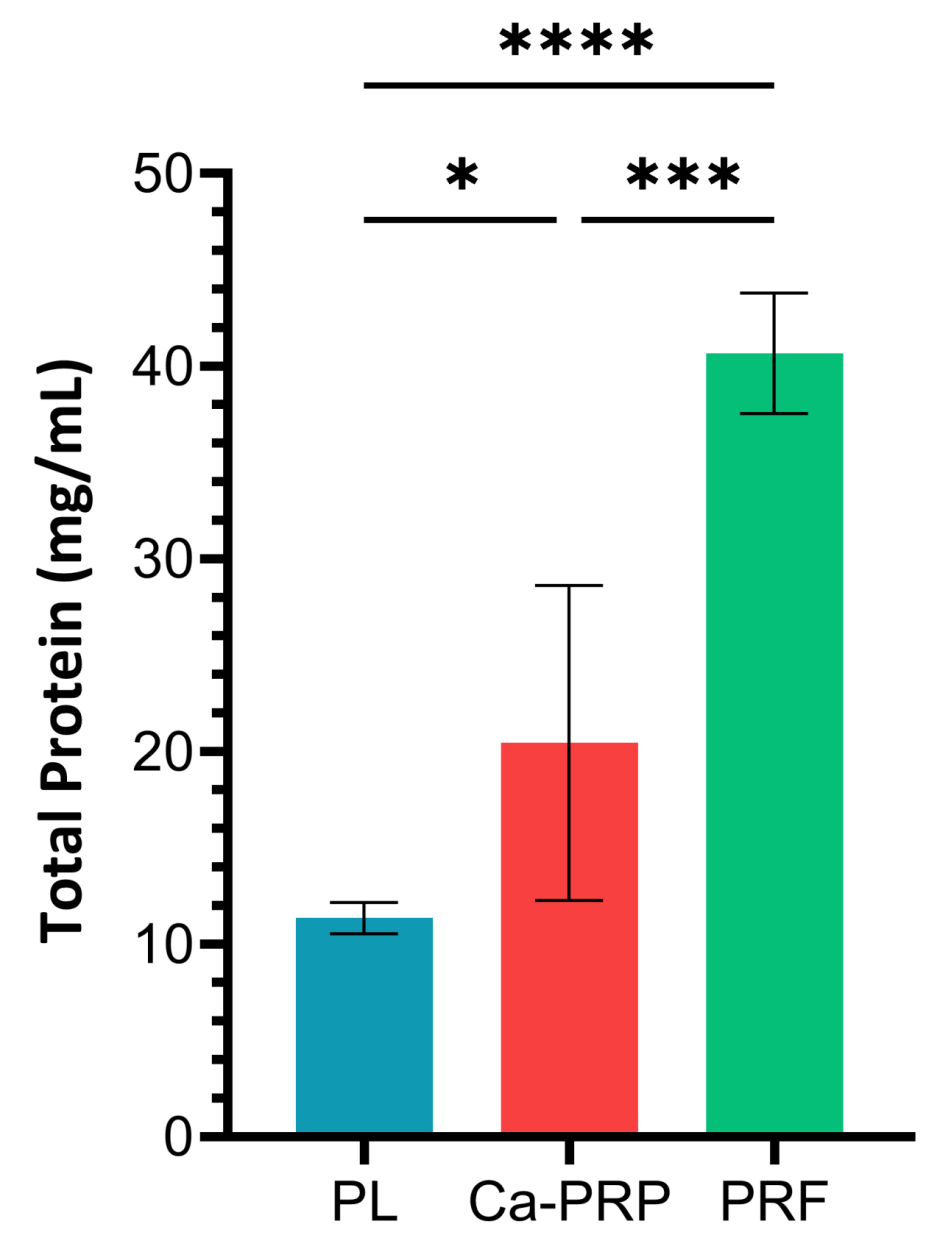
Bradford assay-based quantification of total protein. Values are reported as the mean of 5 samples, with error bars representing standard deviation. Significance determined via Tukey’s post-hoc comparison tests following one-way ANOVA. *p< 0.05, ** p<0.005, *** p<0.0005, **** p<0.00005

PRF was found to have the highest protein content (40.67±3.13 mg/mL), followed by Ca-PRP (20.44±8.17 mg/mL) then PL (11.35±0.80 mg/mL). Statistical significant (p<0.05) was demonstrated for these differences, with it being strongest between PL/PRF (p<0.00005), followed by Ca-PRP/PRF (p<0.0005) then Ca-PRP/PL (p<0.05) as seen in Figure 3 (Fig. 3).

### SDS-PAGE

**Figure 4:**
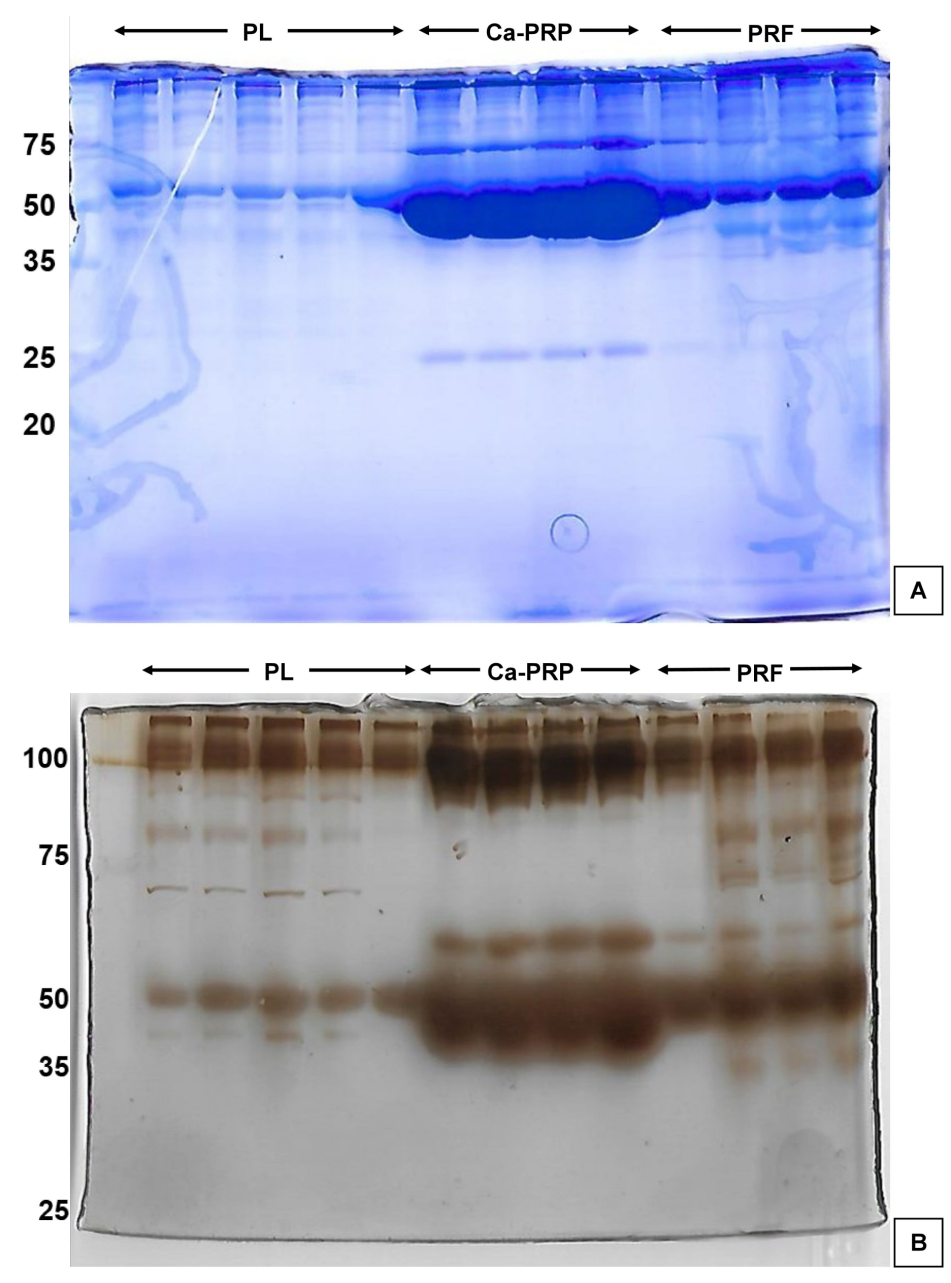
SDS-PAGE gels. A) 12% polyacrylamide running gel, Coomassie blue stain. B) 8% polyacrylamide running gel, silver stain.

Protein zones (Figure 4 (Fig. 4)) were clearly visible at every point of the protein marker (25, 35, 50, 75 and 100 kDa) and between them. The most abundant zone appeared at ~50 kDa, which was most prominent in Ca-PRP and least prominent in PL. Further separation revealed another abundant zone at 60 and 100 kDa, respectively. It is also interesting to note that – where visible – all samples produced the same zones except between ~80 and 50 kDa where a deal of variability can be noted.

Based on their molecular weights (kDa) the six GFs would fall in the following zones: Endostatin, 178.19; EGF, 134; HGF, 83.13; TGF β1, 44.34; VEGF, 22.31; and SDF-1α, 10.67 [35], [36], [37], [38], [39], [40].

### Growth factor (GF) content

GF concentrations are reported in Table 1 (Tab. 1) as the mean (± standard deviation), and in Table 2 (Tab. 2) as a percentage of total protein.

**Figure 5:**
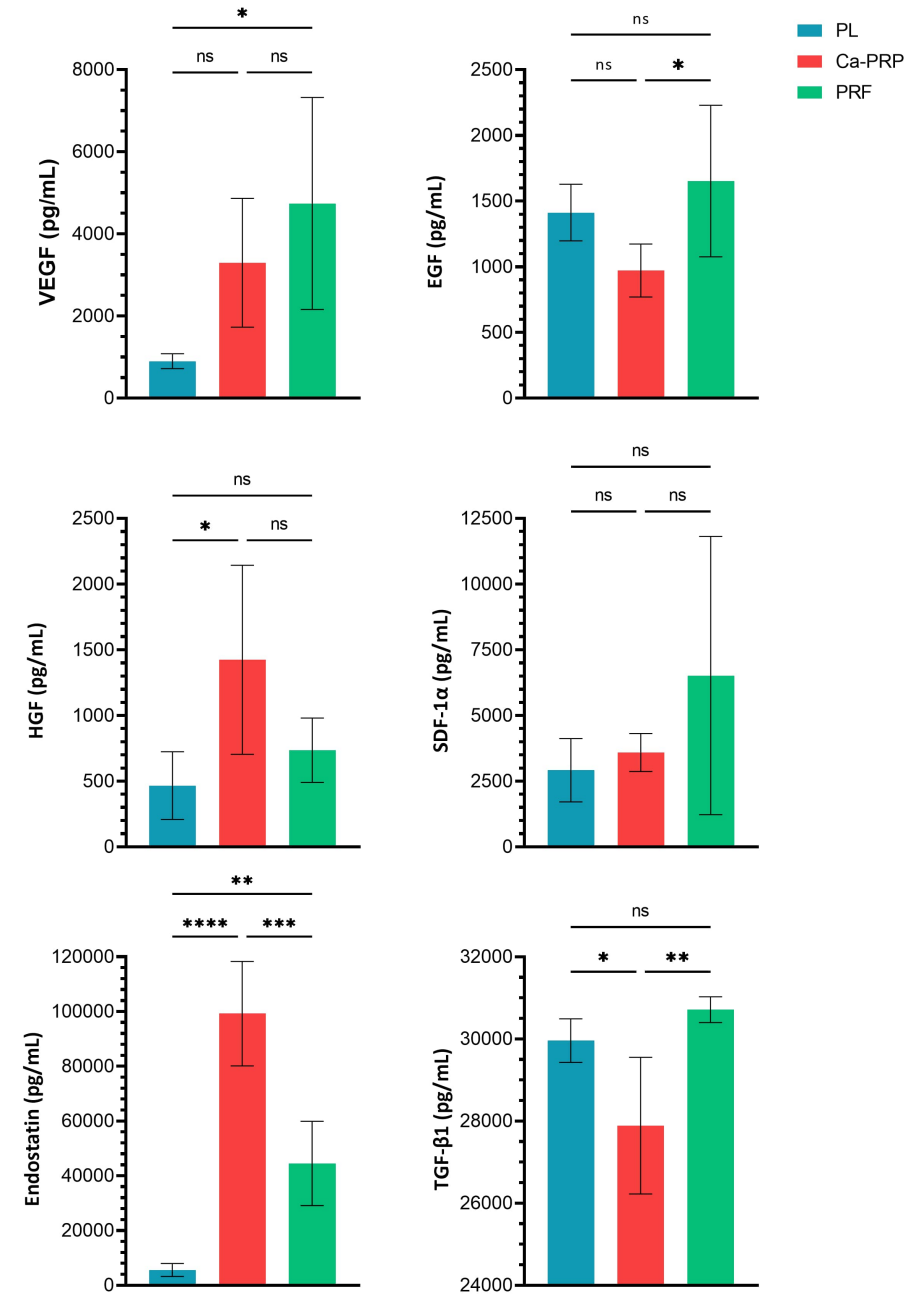
sELISA-based quantification of growth factors. Values are reported as the mean of 5 samples, with error bars representing standard deviation. Significance determined via Tukey’s post-hoc comparison tests following one-way ANOVA. * p<0.05, ** p<0.005, *** p<0.0005, **** p<0.00005

A significant (p<0.05) difference in concentration was demonstrated for five GFs between ≥1 samples as shown in Figure 5 (Fig. 5). However, no statistically significant difference was shown between either sample for SDF-1α (Figure 5 (Fig. 5)).

The pattern (4 of 6 GFs) shows that in general, PL contains the lowest concentration of measured GFs. The exceptions being EDF and TGF-β1, where it was nonetheless surpassed by PRF. On the other end, PRF contains the largest concentration of GFs, except for HGF and endostatin, where it was surpassed by Ca-PRP.

## Discussion and conclusion

As part of a long-term project to compound platelet-derived formulations for various stages in the evolution of a chronic wounds, this study quantified total protein and six angiogenically-active growth factors in three PDPs: PL, Ca-PRP and PRF. While both protein recovery and GF composition varied considerably among the explored PDPs, we demonstrate that all are an abundant source of EGF, VEGF, HGF, SDF-1α, endostatin, and TGF-β1. The data complimented previous work from this laboratory that uncovered substantial amounts of IgG and albumin in the PDPs [22]. It can thus be summarized that, collectively, these molecules protect wounds from even subclinical infection and inflammation, while promoting angiogenesis and healing. The new data further enlightens the platelet proteome.

Platelets are the first elements to arrive at the site of the injury and are particularly active in the early inflammatory phase of the healing process. They play a major role in initiating wound repair by locally releasing several GFs through the α-granules degranulation. They regulate aggregation, clot formation, recruitment of the inflammatory cells and promote tissue repair through the cytokines and proteins released [20], [21].

An ever-growing body of research shows platelets playing key roles in subsequent phases of wound healing, and other physiological and pathological functions such as immunity, diabetes mellitus and atherosclerosis [41]. It has therefore been postulated that autologous GFs derived from circulating platelets may be used for the treatment of chronic wounds. Its application in intractable ankle ulceration in β-thalassemia homozygotes, diabetic foot ulcers, orthopedic injuries and regenerative dentistry are good, albeit anecdotal, examples [9], [10], [11], [12], [13], [14], [15], [16], [17], [18]. The rationale is that application of PDPs to a wound delivers a concentrated, yet physiological mix of bioactive molecules such as GFs and immunoglobulins that can – among other things – resolve chronic inflammation and herald cell proliferation. 

However, to date, there have been no large-scale robust clinical trials. Arguably, this is because such endeavors would require a robust catalogue of PDPs’ biochemical composition, as well as standardization in PDP methodology and nomenclature (e.g. what constitutes PRP) [42] For instance: demographical variation in GF concentration (age, sex, platelet count and physical exertion prior to donation), centrifugation speed (e.g. due to apparatus variations), time from preparation to analysis, and in the case of Ca-PRP and PRF, the concentration and nature of the activating factor [27], [42], [43], [44], [45]. Moreover, it must be noted that platelets are precious clinical material. It is therefore unlikely and unfeasible to procure the necessary quantities for such large-scale trials. However, human in vitro models and an animal source such as porcine platelets, could be appropriate for deeper exploration [46].

The diverse molecular profiles of PDPs raise the possibility of mixing to provide the most needed molecules during the life history of a wound [9]. For instance, impaired angiogenesis in diabetic ulcers leads to a reduced afflux of inflammatory cells and thus, a poor release of cytokines and GFs, and susceptibility to infection [47]. As such, the application of an autologous platelet-derived mix of immunoglobulins and anti-inflammatory GFs could stave off infection and quell inflammation, thus allowing progression to phase 2 of healing. The subsequent application of pro-proliferative GFs could then boost re-epithelization and angiogenesis, to provide maximum therapeutic benefit to the ulcer.

While on their own, PDPs do not appear to be a completely suitable therapeutic, supplementation with one or more of the GFs reported here and elsewhere could serve to compliment the beneficial effects in a stage-specific manner. If successful, the stage-specific mixes could be replaced with cheaper, mass-producible bio-manufactured preparations.

## Notes

### Contributorship 

S.C. collected and processed samples, quantified growth factors, analysed and presented the data and authored the manuscript. B.B. quantified total protein and executed SDS-PAGE. E.S. supervised all lab work and co-conceptualized the project with AF. All authors reviewed and approved the manuscript.

### ORCIDs of the authors

- Scott Custo: 0000-0002-7725-3175
- Byron Baron: 0000-0001-5722-6295
- Alex Felice: 0000-0003-0015-3923
- Elisa Seria: 0000-0002-3003-4151

### Ethics 

This study was approved by the Research Ethics Committee of the Faculty of Medicine and Surgery, University of Malta (FRECMDS_2021_057).

### Funding

This research was supported by a fellowship granted by the Foundation for Medical Service (FMS); Malta Enterprise; and research funds from the Faculty of Medicine and Surgery, University of Malta. Funding reference number MDSRA01–01. The funding bodies played no role in the design of the study and collection, analysis, and interpretation of data and in writing the manuscript.

### Competing interests

The authors declare that they have no competing interests. Part of this work was submitted to the University of Malta by S.C., in part-fulfillment of the degree of Bachelor of Science (Honors) in Medical Sciences.

### Acknowledgements

The authors would like to thank the Malta National Blood Transfusion Service (Guardamangia, Malta), specifically Dr. Vanessa Zammit, for their much-appreciated help in obtaining the samples for this project.

## Figures and Tables

**Table 1:**
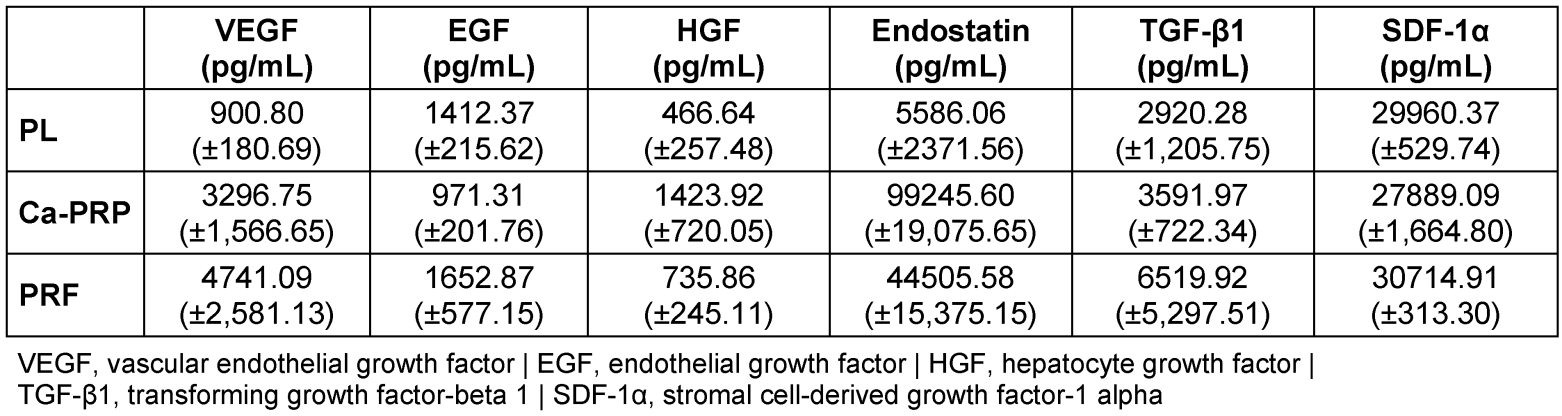
Growth factor content (pg/mL) of platelet lysate (PL), calcium-activated platelet-rich plasma (Ca-PRP) and platelet-rich fibrin (PRF), determined using sandwich enzyme-linked immunosorbent assay. Values are reported as the mean of 5 samples (± standard deviation).

**Table 2:**
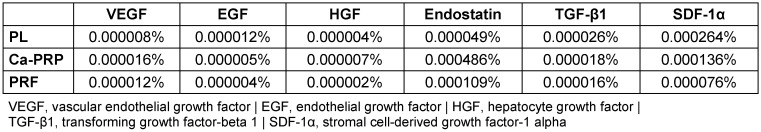
Growth factor content as a percentage of total protein in platelet lysate (PL), calcium-activated platelet-rich plasma (Ca-PRP) and platelet-rich fibrin (PRF). GF content determined using sandwich enzyme-linked immunosorbent assay, total protein determined via the Bradford Assay.

## References

[1] Velnar T, Bailey T, Smrkolj V (2009). The wound healing process: an overview of the cellular and molecular mechanisms. J Int Med Res.

[2] Frykberg RG, Banks J (2015). Challenges in the Treatment of Chronic Wounds. Adv Wound Care (New Rochelle).

[3] Guest JF, Vowden K, Vowden P (2017). The health economic burden that acute and chronic wounds impose on an average clinical commissioning group/health board in the UK. J Wound Care.

[4] Trøstrup H, Thomsen K, Christophersen LJ, Hougen HP, Bjarnsholt T, Jensen PØ, Kirkby N, Calum H, Høiby N, Moser C (2013). Pseudomonas aeruginosa biofilm aggravates skin inflammatory response in BALB/c mice in a novel chronic wound model. Wound Repair Regen.

[5] Roche ED, Renick PJ, Tetens SP, Ramsay SJ, Daniels EQ, Carson DL (2012). Increasing the presence of biofilm and healing delay in a porcine model of MRSA-infected wounds. Wound Repair Regen.

[6] Guest JF, Ayoub N, McIlwraith T, Uchegbu I, Gerrish A, Weidlich D, Vowden K, Vowden P (2015). Health economic burden that wounds impose on the National Health Service in the UK. BMJ Open.

[7] Guest JF, Ayoub N, McIlwraith T, Uchegbu I, Gerrish A, Weidlich D, Vowden K, Vowden P (2017). Health economic burden that different wound types impose on the UK’s National Health Service. Int Wound J.

[8] Guest JF, Fuller GW, Vowden P (2020). Cohort study evaluating the burden of wounds to the UK’s National Health Service in 2017/2018: update from 2012/2013. BMJ Open.

[9] Josifova D, Gatt G, Aquilina A, Serafimov V, Vella A, Felice A (2001). Treatment of leg ulcers with platelet-derived wound healing factor (PDWHFS) in a patient with beta thalassaemia intermedia. Br J Haematol.

[10] Gilsanz F, Escalante F, Auray C, Olbés AG (2001). Treatment of leg ulcers in beta-thalassaemia intermedia: use of platelet-derived wound healing factors from the patient's own platelets. Br J Haematol.

[11] Wu PI, Diaz R, Borg-Stein J (2016). Platelet-Rich Plasma. Phys Med Rehabil Clin N Am.

[12] Singh SP, Kumar V, Pandey A, Pandey P, Gupta V, Verma R (2018). Role of platelet-rich plasma in healing diabetic foot ulcers: a prospective study. J Wound Care.

[13] Elsaid A, El-Said M, Emile S, Youssef M, Khafagy W, Elshobaky A (2020). Randomized Controlled Trial on Autologous Platelet-Rich Plasma Versus Saline Dressing in Treatment of Non-healing Diabetic Foot Ulcers. World J Surg.

[14] Döri F, Arweiler N, Húszár T, Gera I, Miron RJ, Sculean A (2013). Five-year results evaluating the effects of platelet-rich plasma on the healing of intrabony defects treated with enamel matrix derivative and natural bone mineral. J Periodontol.

[15] Miron RJ, Zucchelli G, Pikos MA, Salama M, Lee S, Guillemette V, Fujioka-Kobayashi M, Bishara M, Zhang Y, Wang HL, Chandad F, Nacopoulos C, Simonpieri A, Aalam AA, Felice P, Sammartino G, Ghanaati S, Hernandez MA, Choukroun J (2017). Use of platelet-rich fibrin in regenerative dentistry: a systematic review. Clin Oral Investig.

[16] Roselló-Camps À, Monje A, Lin GH, Khoshkam V, Chávez-Gatty M, Wang HL, Gargallo-Albiol J, Hernandez-Alfaro F (2015). Platelet-rich plasma for periodontal regeneration in the treatment of intrabony defects: a meta-analysis on prospective clinical trials. Oral Surg Oral Med Oral Pathol Oral Radiol.

[17] Miron RJ, Fujioka-Kobayashi M, Bishara M, Zhang Y, Hernandez M, Choukroun J (2017). Platelet-Rich Fibrin and Soft Tissue Wound Healing: A Systematic Review. Tissue Eng Part B Rev.

[18] Xia Y, Zhao J, Xie J, Lv Y, Cao DS (2019). The Efficacy of Platelet-Rich Plasma Dressing for Chronic Nonhealing Ulcers: A Meta-Analysis of 15 Randomized Controlled Trials. Plast Reconstr Surg.

[19] Ma L, Elliott SN, Cirino G, Buret A, Ignarro LJ, Wallace JL (2001). Platelets modulate gastric ulcer healing: role of endostatin and vascular endothelial growth factor release. Proc Natl Acad Sci USA.

[20] Opneja A, Kapoor S, Stavrou EX (2019). Contribution of platelets, the coagulation and fibrinolytic systems to cutaneous wound healing. Thromb Res.

[21] Golebiewska EM, Poole AW (2015). Platelet secretion: From haemostasis to wound healing and beyond. Blood Rev.

[22] Seria E, Samut Tagliaferro S, Cutajar D, Galdies R, Felice A (2021). Immunoglobulin G in Platelet-Derived Wound Healing Factors. Biomed Res Int.

[23] George JN, Saucerman S, Levine SP, Knieriem LK, Bainton DF (1985). Immunoglobulin G is a platelet alpha granule-secreted protein. J Clin Invest.

[24] Oka Y, Orth DN (1983). Human plasma epidermal growth factor/beta-urogastrone is associated with blood platelets. J Clin Invest.

[25] Möhle R, Green D, Moore MA, Nachman RL, Rafii S (1997). Constitutive production and thrombin-induced release of vascular endothelial growth factor by human megakaryocytes and platelets. Proc Natl Acad Sci USA.

[26] Webb NJ, Bottomley MJ, Watson CJ, Brenchley PE (1998). Vascular endothelial growth factor (VEGF) is released from platelets during blood clotting: implications for measurement of circulating VEGF levels in clinical disease. Clin Sci (Lond).

[27] Taniguchi Y, Yoshioka T, Sugaya H, Gosho M, Aoto K, Kanamori A, Yamazaki M (2019). Growth factor levels in leukocyte-poor platelet-rich plasma and correlations with donor age, gender, and platelets in the Japanese population. J Exp Orthop.

[28] Xu X, Zhu F, Zhang M, Zeng D, Luo D, Liu G, Cui W, Wang S, Guo W, Xing W, Liang H, Li L, Fu X, Jiang J, Huang H (2013). Stromal cell-derived factor-1 enhances wound healing through recruiting bone marrow-derived mesenchymal stem cells to the wound area and promoting neovascularization. Cells Tissues Organs.

[29] Stellos K, Langer H, Daub K, Schoenberger T, Gauss A, Geisler T, Bigalke B, Mueller I, Schumm M, Schaefer I, Seizer P, Kraemer BF, Siegel-Axel D, May AE, Lindemann S, Gawaz M (2008). Platelet-derived stromal cell-derived factor-1 regulates adhesion and promotes differentiation of human CD34+ cells to endothelial progenitor cells. Circulation.

[30] Massberg S, Konrad I, Schürzinger K, Lorenz M, Schneider S, Zohlnhoefer D, Hoppe K, Schiemann M, Kennerknecht E, Sauer S, Schulz C, Kerstan S, Rudelius M, Seidl S, Sorge F, Langer H, Peluso M, Goyal P, Vestweber D, Emambokus NR, Busch DH, Frampton J, Gawaz M (2006). Platelets secrete stromal cell-derived factor 1alpha and recruit bone marrow-derived progenitor cells to arterial thrombi in vivo. J Exp Med.

[31] Battinelli EM, Markens BA, Italiano JE (2011). Release of angiogenesis regulatory proteins from platelet alpha granules: modulation of physiologic and pathologic angiogenesis. Blood.

[32] Assoian RK, Komoriya A, Meyers CA, Miller DM, Sporn MB (1983). Transforming growth factor-beta in human platelets. Identification of a major storage site, purification, and characterization. J Biol Chem.

[33] Assoian RK, Sporn MB (1986). Type beta transforming growth factor in human platelets: release during platelet degranulation and action on vascular smooth muscle cells. J Cell Biol.

[34] Lee HM, Shen EC, Shen JT, Fu E, Chiu HC, Hsia YJ (2020). Tensile strength, growth factor content and proliferation activities for two platelet concentrates of platelet-rich fibrin and concentrated growth factor. J Dent Sci.

[35] UniProtKB – P15692 (VEGFA_HUMAN).

[36] UniProtKB – P39060 (COIA1_HUMAN).

[37] UniProtKB – P01133 (EGF_HUMAN).

[38] UniProtKB – P14210 (HGF_HUMAN).

[39] UniProtKB – P01137 (TGFB1_HUMAN).

[40] UniProtKB – P48061 (SDF1_HUMAN).

[41] Michelson A, Cattaneo M, Frelinger A, Newman P (2019). Platelets.

[42] Durante C, Agostini F, Abbruzzese L, Toffola RT, Zanolin S, Suine C, Mazzucato M (2013). Growth factor release from platelet concentrates: analytic quantification and characterization for clinical applications. Vox Sang.

[43] Fréchette JP, Martineau I, Gagnon G (2005). Platelet-rich plasmas: growth factor content and roles in wound healing. J Dent Res.

[44] Hamilton B, Tol JL, Knez W, Chalabi H (2015). Exercise and the platelet activator calcium chloride both influence the growth factor content of platelet-rich plasma (PRP): overlooked biochemical factors that could influence PRP treatment. Br J Sports Med.

[45] Cavallo C, Roffi A, Grigolo B, Mariani E, Pratelli L, Merli G, Kon E, Marcacci M, Filardo G (2016). Platelet-Rich Plasma: The Choice of Activation Method Affects the Release of Bioactive Molecules. Biomed Res Int.

[46] Seria E, Galea G, Borg J, Schembri K, Grech G, Tagliaferro SS, Felice A (2021). Novel leukocyte-depleted platelet-rich plasma-based skin equivalent as an in vitro model of chronic wounds: a preliminary study. BMC Mol Cell Biol.

[47] Honnegowda TM, Kumar P, Udupa EG, Kumar S, Kumar U, Rao P (2015). Role of angiogenesis and angiogenic factors in acute and chronic wound healing. Plast Aesthet Res.

